# Importance of tear volume for positivity of tear matrix metalloproteinase-9 immunoassay

**DOI:** 10.1371/journal.pone.0235408

**Published:** 2020-07-10

**Authors:** Jong Hwa Jun, You Hyun Lee, Myeong Jin Son, Harim Kim

**Affiliations:** Department of Ophthalmology, Dongsan Medical Center, Keimyung University School of Medicine, Daegu, Korea; Universidad Francisco de Vitoria, SPAIN

## Abstract

The tear matrix metalloproteinase-9 (MMP-9) immunoassay (Inflammadry) exhibits variable results in dry eye (DE) patients. We investigated if the tear volume in DE patients affects the results of MMP-9 immunoassay in clinical and *in vitro* settings. This cross-sectional study enrolled 188 eyes of 188 DE patients. The clinical symptoms and signs of DE were assessed using the Ocular Surface Disease Index and visual analog scale, strip meniscometry, tear break-up time, and tear meniscus height (TMH), area (TMA), and depth (TMD) using swept-source optical coherence tomography and corneal and conjunctival staining scores. For quantitative evaluation, the bands produced by the InflammaDry test were analyzed with ImageJ. DE subjects were grouped according to MMP-9 positivity and TMH. The InflammaDry-positive group showed greater TMH, TMA, and TMD than the MMP-9-negative group (p < 0.05). InflammaDry test band density in the high TMH group was significantly greater than that in the low and normal TMH groups (p < 0.05). InflammaDry test band density correlated positively with TMH, TMA, and TMD (all p < 0.05). InflammaDry test results were influenced by tear volume. Low tear volume in aqueous tear-deficient DE may induce false-negative results, and reflex tearing during the test may induce false-positive results.

## Introduction

Dry eye (DE) is a multifactorial disease caused by changes in the quality and/or quantity of the precorneal tear film [1]. Current diagnosis of DE is based on a combination of clinical signs, such as those determined by the Schirmer test, tear break-up time (tBUT), and staining scores, as well as symptoms, which are determined by formalized questionnaires. Although the Schirmer test results, tBUT, and corneal staining scores have long been used as the main indicators of DE, these tests lack objectivity and show low reproducibility due to user-dependent errors [2,3]. Therefore, efforts are being made to develop new objective methods for DE diagnosis, such as tear osmolarity tests and the tear matrix metalloproteinase (MMP)-9 immunoassay [4,5].

MMP-9 plays two key roles in ocular pathophysiology. In tears, MMP-9 expression is normally below 40 ng/mL and is secreted from the ocular surface epithelium [6]. MMP-9 maintains the epithelial barrier function by cleaving epithelial basement membrane components and tight-junction proteins, such as ZO-1 and occludin [7,8]. On the other hand, MMP-9 is also related to the pathogenesis of various diseases, such as sterile ulceration, ocular allergy, keratoconus, conjunctivochalasis, and DE [3,9–11]. In the pathology of DE, from the initiation of tear hyperosmolarity in the early phase of DE, a vicious cycle of DE likely induces inflammatory processes and triggers the release of MMP-9 in a relatively late phase of DE [12]. As detection of elevated tear MMP-9 could be an ideal tool for diagnosis and management of DE, a tear MMP-9 immunoassay could be valuable to clinicians [13].

Most studies have reported superior sensitivity and specificity of MMP-9 immunoassay compared to conventional diagnostics, and the MMP-9 immunoassay reflected the clinical signs and symptoms well in those reports [5,6,14]. However, Lanza et al. failed to find differences in clinical symptoms, underlying diseases, and clinical signs of DE between MMP-9-positive and -negative groups [15,16]. In addition, although not statistically significant, Schargus et al. showed that the Schirmer score was higher in the MMP-9-positive group than in the -negative group [3]. Further, a recent *in vitro* study that investigated the pre-form and active-form of human MMP-9 in a commercially available MMP-9 immunoassay revealed dependency of the result on the loading volume [17]. Thus, not only severely tear-deficient DE conditions, such as Sjögren syndrome and ocular graft-versus-disease, but also mild to moderate aqueous tear-deficient DE may produce false-negative results.

Herein, we aimed to elucidate the impact of tear volume on positivity in a commercially available MMP-9 immunoassay (Inflammadry, Quidel Corporation, San Diego, CA, USA) in DE patients and investigated changes in MMP-9 immunoassay-positivity according to the tear volume, as measured by swept-source optical coherence tomography (OCT).

## Materials and methods

### Study population and setting

This study was conducted in accordance with the ethical principles of the Declaration of Helsinki. The study protocol and written informed consent were approved by the Keimyung University Dongsan Medical Center Institutional Review Board (IRB no. DSMC 2017-06-008-005). This cross-sectional study was performed in DE patients who visited our ophthalmic department from April 1, 2017, through June 30, 2019. Written informed consent was obtained from all adult patients and two patients under 19 received consent from their parents after a detailed explanation of the study was provided. The study investigator collected clinical data and MMP-9 immunoassay (InflammDry) results from the right eye of each enrolled patient to avoid a coupling effect.

The clinical diagnosis of DE was made according to the following three criteria in the right eye of each patient: Ocular Surface Disease Index (OSDI) score of more than 12, tBUT of less than 10 s, and corneal fluorescein staining results of 1 or more by the Oxford scheme. The patients who met all the three criteria were classified as having DE. Exclusion criteria were as follows: (1) active or recent keratitis or conjunctivitis within the previous 4 weeks, (2) moderate to severe conjunctivochalasis, (3) any stage of keratoconus, (4) lacrimal drainage disorders, such as lacrimal punctal stenosis, deformed lacrimal punctum, canalicular anomalies, and nasolacrimal duct obstruction, or temporary or permanent punctal occlusion, (5) topical or systemic corticosteroid treatment or immunomodulatory therapy within the previous 4 weeks, (6) fluorescein, cornstarch, or Dacron allergy, (7) undergone ocular surgery within the previous 6 months, or had ocular trauma in the previous 3 months, and (8) use of contact lenses within the previous 72 h.

### Clinical assessment

Clinical examinations were performed in the following order: tear meniscus measurements by anterior swept-source OCT, OSDI questionnaire/visual analog scale (VAS), tear MMP-9 immunoassay (InflammaDry test), strip meniscometry (SM tube^®^, Echo Electricity Co., Ltd., Fukushima, Japan), tBUT, and both corneal and conjunctival staining scores by fluorescein staining.

**Fig 1:**
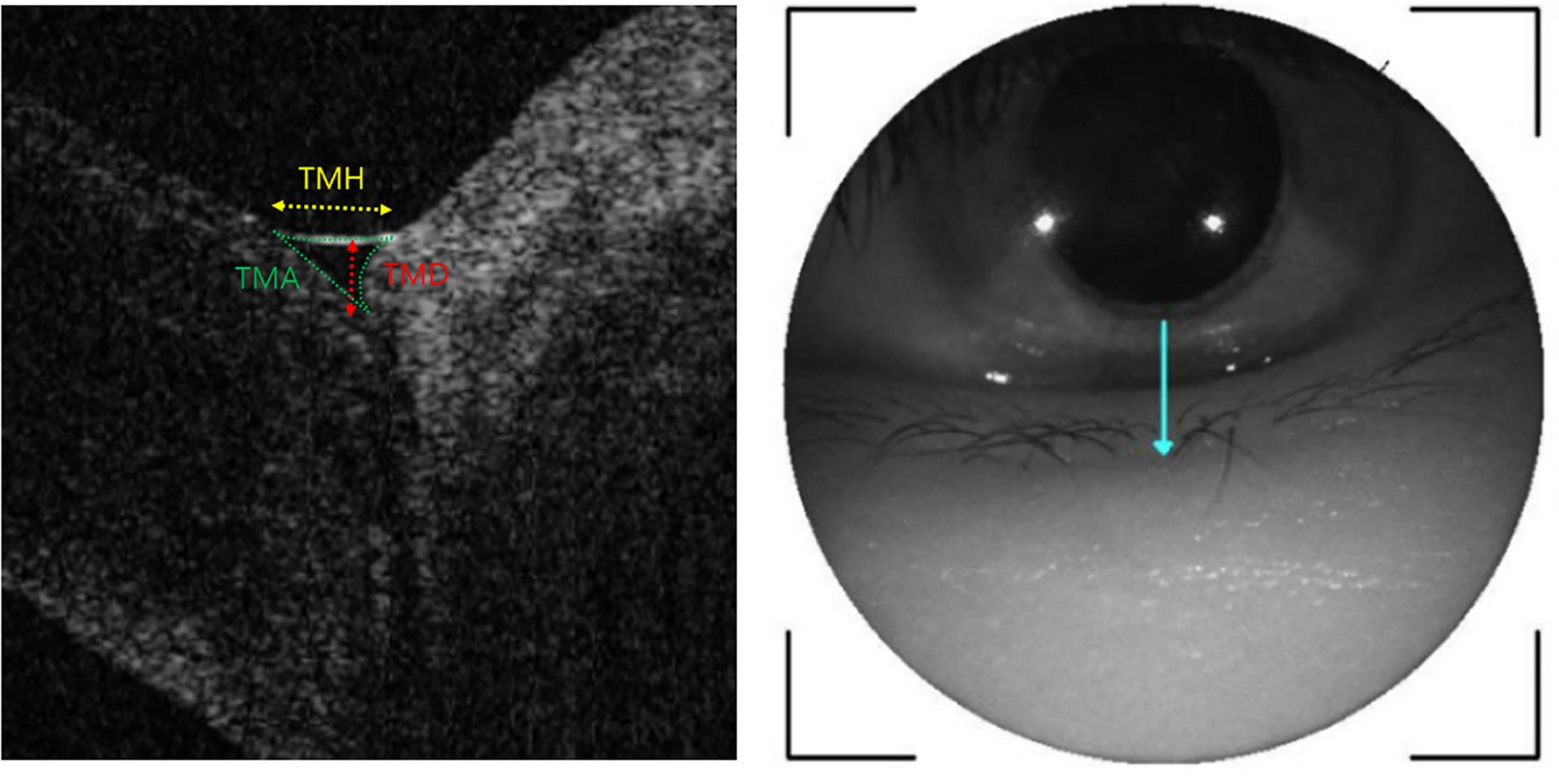
Measurements of TMH, TMD, and TMA. TMH (yellow line) was measured as the distance from the cornea–meniscus junction to the lower eyelid–meniscus junction. TMD (red line) was measured as the vertical distance from the interface of the cornea with the lower eyelid to the yellow line. TMA (area bounded by green lines) was calculated proportionally using ImageJ software from the OCT measured TMH value, TMA was calculated by the square of the ratio multiplied by the ImageJ-based TMA.

The anterior segment OCT image was acquired with a corneal module mounted on a swept-source OCT device (DRI OCT Triton; Topcon, Tokyo, Japan). All tear parameters were measured by a single experienced technician. Participants were instructed to blink three times, followed by gazing straight forward, and the reference was aligned to the inferior cornea and the center of the lower eyelid. A single vertical raster image of the right eye was obtained 3 s after voluntary blinking in a dark room. Tear meniscus height (TMH) was measured as the distance between the cornea–meniscus junction and the lower eyelid–meniscus junction. Tear meniscus depth (TMD) was taken as the vertical distance from the interface of the cornea with the lower eyelid to the TMH line. Tear meniscus area (TMA) was calculated proportionally using ImageJ software (ImageJ 1.44p; National Institutes of Health, Bethesda, MD, USA) [18,19]. First, the anterior segment OCT image with the measured TMH value was captured. Then, the captured image was mounted to the ImageJ program and the TMH and TMA were measured. After the ratio of the ImageJ-based TMH to the anterior segment OCT-based TMH was obtained, the anterior segment OCT-based TMA was calculated by the square of the ratio multiplied by the ImageJ-based TMA (Fig 1).

Strip meniscometry was performed using a commercially available kit composed of a polyethylene terephthalate panel and centrally embedded 8 um nitrocellulose membrane filter paper strip. The strip was positioned adjacent to the right lower palpebral conjunctiva and the examiner tried to absorb the tear lake of each participant during 5 seconds. Tear volume was estimated based on the length of wetted strip.

After tear meniscus measurements, participants’ symptoms were evaluated using the OSDI questionnaire [20]. This consisted of 12 questions on eye-related symptoms, vision-related function, and environment-related symptoms. The scores range from 0 to 100, where 0 indicates no discomfort, and 100 indicates the most severe symptom and disability. In addition, the severity of ocular pain was analyzed using a VAS, where 0 indicates no pain, and 10 indicates the worst possible pain [21].

The tear MMP-9 immunoassay (InflammaDry test) was performed according to the manufacturer’s instruction by a single examiner (JHJ). The sampling fleece was contacted three times at each location of the inferior palpebral conjunctival (temporal, middle, nasal; from nasal to temporal direction) and was rested against the temporal inferior palpebral conjunctiva for an additional 5 s. After assembling the sampling fleece to a sample collector, the test strip was dipped into a buffer solution for 20 s. To evaluate the result-band’s density, the result window was photographed with a slit-lamp biomicroscope-mounted single-lens reflex camera (Canon EOS 700D, setting: ISO 400, shutter speed 1/200 s) at 10 min after test initiation. For quantitative evaluation of the result band of the InflammaDry test, band densitometry was performed with ImageJ. Qualitative evaluations (positive/negative) were also performed by a single examiner (JHJ), and faint to strong bands were judged as positive, and the absence of a band was considered as negative, according to the manufacturer’s instruction.

tBUT was evaluated after touching a fluorescein strip wetted with normal saline (Haag-Streit AG, Koenig, Switzerland) to the lower inferotemporal palpebral conjunctiva. After blinking three times, the interval from the last blink to the first appearance of dark spots on the corneal surface was measured using a stopwatch.

Corneal and conjunctival staining was performed using fluorescein stain, and the score was measured under the Oxford grading system. The staining score ranges from 0 to 5 for each panel and from 0 to 15 for the total exposed interpalpebral conjunctiva and cornea [22]. The conjunctival stain score was also checked at both the nasal and temporal sides of the right eye.

### Grouping of participants according to TMH and InflammaDry-positivity

To evaluate the influence of tear volume on InflammaDry test positivity, we grouped the participants according to InflammaDry positivity and the TMH value measured by anterior OCT. According to the results of the Inflammadry test, we divided the DE patients into InflammaDry-positive and -negative groups. DE patients were also allocated to three groups (low, normal, and high) according to their TMH results. A TMH value of 77 to 210 μm was indicated as a low TMH, 211 to 310 μm as a normal TMH, and over 311 μm as high TMH.

### Statistical analysis

Data are expressed as means ± standard deviation (SD) unless otherwise specified. Statistical analyses were performed using SPSS version 12.0 (IBM, Chicago, IL, USA). The between-group differences in age, OSDI/VAS score, tBUT, corneal or conjunctival staining, TMH, TMD, and TMA were compared using independent t-tests and one-way analysis of variance. Post-hoc tests of between-group analyses were performed using the Tukey HSD test. Pearson’s correlation test was performed between the MMP-9 assay band densitometry results and the OSDI/VAS score, tBUT, corneal or conjunctival staining score, TMH, TMD, and TMA. *P* values of 0.05 or less were considered statistically significant.

## Results

### Demographics of study populations

One-hundred-and-eighty-eight DE patients (188 eyes) were enrolled in the study. The patients’ mean age was 58.8 ± 13.0 years (range: 17–86 years), and 47 (33.3%) were men. Positive MMP-9 tests were confirmed in 120 patients, and negative results were noted in 68 patients. There were 64 patients in the low TMH group, 76 in the normal TMH group, and 48 in the high TMH group. Demographic data of patients according to InflammaDry-positivity and TMH measurements are shown in Table 1.

**Table 1:** Patient demographics of TMH groups and InflammaDry positivity groups.

	**Low TMH (n = 64)**	**Normal TMH (n = 76)**	**High TMH (n = 48)**	***p*-value**
Age (mean ± SD), years	58.3 ± 11.2	56.9 ± 15.2	62.4 ± 10.9	0.177
Sex (M/F)	9/55	18/58	20/28	**0.004**
Diabetes (Y/N)	4/60	7/69	1/47	0.286
Hypertension (Y/N)	10/54	20/56	19/29	**0.017**
Sjögren syndrome (Y/N)	7/57	12/64	3/45	0.266

	**InflammaDry-positive (n = 120)**	**InflammaDry-negative (n = 68)**	***p*-value**
Age (mean ± SD), years	59.2 ± 13.2	58.0 ± 12.8	0.548
Sex (M/F)	35/85	12/56	0.080
Diabetes (Y/N)	10/110	2/66	0.146
Hypertension (Y/N)	36/84	13/55	0.102
Sjögren syndrome (Y/N)	17/103	5/63	0.163

TMH = tear meniscus height, SD = standard deviation, Y/N = Yes/No.

### Comparisons of clinical parameters between the three TMH groups

There was no difference in OSDI/VAS and strip meniscometry results between the three TMH groups. tBUT differed significantly among the three TMH groups in the post-hoc test (low vs high TMH group: p = 0.012; normal vs high TMH group: p = 0.024). In the high TMH group, tBUT was longer than that in the low TMH group. Corneal and conjunctival staining scores showed significant differences among the three TMH groups (Table 2). The corneal staining score showed significant differences between the low and high TMH groups (p = 0.014) and between the normal and high TMH groups (p = 0.029). The staining score of both the nasal and temporal conjunctiva in the low TMH group was higher than those in the high TMH groups (p = 0.014 and 0.013, respectively). There was no difference between the low and normal TMH, or normal and high TMH, in corneal and conjunctival staining.

**Table 2:** Comparisons of clinical parameters within three TMH groups.

Groups	Low TMH (n = 64)	Normal TMH (n = 76)	High TMH (n = 48)	*p*-value
OSDI	37.3 ± 25.9	38.4 ± 20.0	40.9 ± 25.8	0.720
VAS	3.1 ± 3.2	3.5 ± 2.4	3.1 ± 2.9	0.550
Strip meniscometry (mm)	3.7 ± 1.7	4.3 ± 2.1	4.2 ± 2.2	0.155
tBUT (s)	3.2 ± 2.3	3.4 ± 1.7	4.4 ± 2.2	**0.009**
Ocular staining scores, grade				
Corneal	1.6 ± 1.1	1.5 ± 0.9	1.1 ± 0.9	**0.011**
Conjunctival, nasal	1.3 ± 1.3	1.0 ± 1.0	0.8 ± 0.8	**0.016**
Conjunctival, temporal	1.0 ± 1.2	0.8 ± 0.9	0.5 ± 0.6	**0.017**
Tear volume parameters				
TMH, μm	169.3 ± 29.9	259.7 ± 30.2	429.9 ± 115.0	**<0.001**
TMA, mm^2^	0.0071 ± 0.0066	0.0163 ± 0.0052	0.0408 ± 0.0223	**<0.001**
TMD, μm	106.6 ± 42.8	165.1 ± 48.0	243.8 ± 73.6	**<0.001**
InflammaDry test band density (AU)	3375.7 ± 3808.9	3029.7 ± 3618.4	5876.2 ± 6206.7	**0.002**

OSDI: Ocular Surface Disease Index, VAS: Visual analogue scale, tBUT: tear break-up time, TMH: tear meniscus height, TMA: tear meniscus area, TMD: tear meniscus depth, AU: arbitrary unit, data are expressed as the mean ± standard deviation.

**Fig 2:**
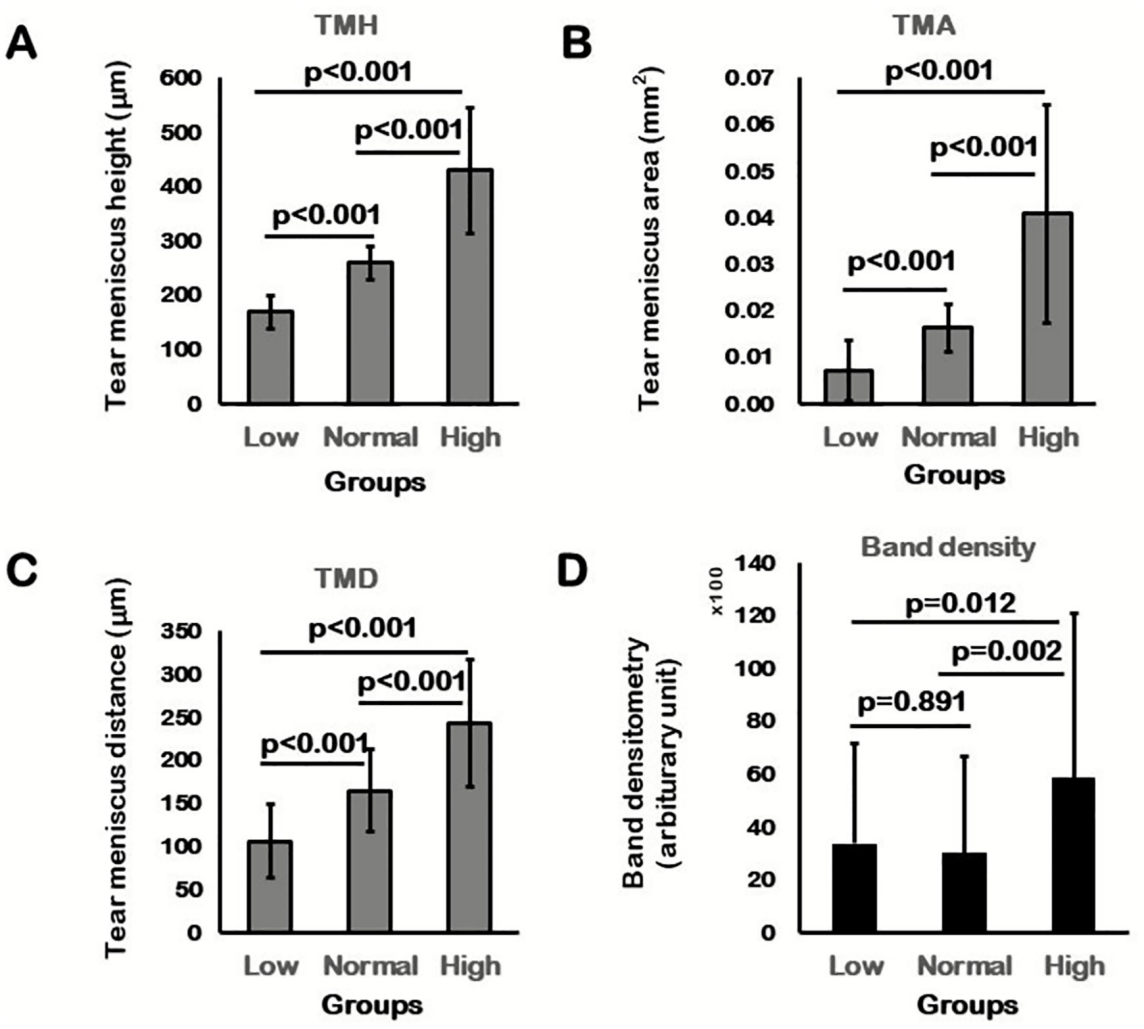
Post-hoc analysis of the three TMH groups. TMH (A), TMA (B), and TMD (C) showed statistically significant differences within the three TMH groups. D. Matrix metalloproteinase (MMP)-9 band density analysis of the three TMH groups showed significantly higher band density in the high TMH group than that in the low and normal TMH groups.

Tear volume measurements by assessing TMH, TMD, and TMA differed significantly in post-hoc tests among the three TMH groups (p < 0.001; Fig 2A–2C). In the comparisons of InflammaDry band density, there were significant differences between the low and high TMH groups (p = 0.012) and between the normal and high TMH groups (p = 0.002). The mean band density was markedly higher in the high TMH group than that in both the low and normal TMH groups (Table 2, Fig 2D).

### Comparisons between InflammaDry-positive and -negative groups

There were no significant differences in ODSI/VAS, strip meniscometry, tBUT, and conjunctival/corneal staining scores between the InflammaDry-positive and -negative groups (p > 0.05). In comparisons between the InflammaDry-positive and -negative groups, there were significant differences in terms of TMH, TMA, and TMD (p = 0.033, 0.017, and 0.010, respectively). The positive group had greater TMH, TMA, and TMD values than the negative group (Table 3).

**Table 3:** Comparisons of clinical characteristics between InflammaDry-positive and -negative groups of dry eye patients.

Groups	InflammaDry-positive (n = 120)	InflammaDry-negative (n = 68)	*p*-value
OSDI	38.3 ± 23.2	39.3 ± 24.5	0.774
VAS	3.4 ± 2.7	3.0 ± 2.9	0.346
tBUT (s)	3.4 ± 1.8	3.9 ± 2.5	0.065
Strip meniscometry (mm)	4.1 ± 2.1	3.9 ± 1.9	0.484
Ocular staining scores, grade			
Corneal	1.5 ± 1.0	1.4 ± 0.9	0.422
Conjunctival, nasal	1.1 ± 1.0	1.0 ± 1.2	0.725
Conjunctival, temporal	0.8 ± 0.9	0.7 ± 0.9	0.156
Tear volume parameters			
TMA, mm^2^	0.0215 ± 0.0196	0.0158 ± 0.0129	**0.017**
TMH, μm	285.1 ± 128.6	249.8 ± 95.3	**0.033**
TMD, μm	175.2 ± 80.6	147.8 ± 61.5	**0.010**

OSDI: Ocular Surface Disease Index, VAS: visual analogue scale, tBUT: tear break-up time, TMH: tear meniscus height, TMA: tear meniscus area, TMD: tear meniscus depth, data are expressed as the mean ± standard deviation.

### Correlation between InflammaDry band density and tear meniscus parameters and clinical signs/symptoms

Pearson’s correlation analysis revealed that InflammaDry band density and tear meniscus parameters, such as TMH, TMA, and TMD, were positively correlated (Table 4). The OSDI/VAS score, tBUT, and corneal or conjunctival staining score showed no correlation with band density (p > 0.05, respectively).

**Table 4:** Correlation analysis between tear meniscus parameters (Height, Depth, and Area) and InflammaDry test band density.

Parameters	TMH	TMD	TMA
r-value	0.218	0.243	0.216
*p*-value	**0.003**	**0.001**	**0.003**

TMA: tear meniscus area, TMD: tear meniscus depth, TMH: tear meniscus height.

## Discussion

MMP-9 is considered a nonspecific and late phase inflammatory marker in DE patients [23–25]. On this basis, an immunoassay was developed to detect and confirm the elevation of tear MMP-9 in DE patients, and recent validation studies reported good diagnostic performance and correlation with clinical severity of DE [5,6,14]. Among these reports, Sambursky et al. reported that this test had 85% sensitivity and 94% specificity for diagnosing DE [14]. However, two separate reports from Schargus et al. and Lanza et al. showed moderate to low positivity in the MMP-9 immunoassay in DE study subjects [3,15,16]. In addition, Lanza et al. reported no difference in subjective symptoms and clinical signs of DE between MMP-9-positive and -negative groups [15,16].

Inflammadry, a point-of-care MMP-9 immunoassay, is based on the principle of lateral-flow immunoassay (LFIA); this type of assay has advantages of low cost, rapid analysis, and ease of device preparation. However, in LFIAs, if the sample volume is small, the reliability of the test result may be significantly impaired. Therefore, despite these advantages, performance of LFIA could be seriously affected by the volume of the loaded analytes [26]. On this basis, in the Inflammadry kit instructions, the manufacturer has also pointed out that less than 6 μl of sample volume could produce false-negative results.

In the present study, there was a tendency for participants having higher tear volumes to show higher band densities, but the subjects who had lower tear volumes, indicating aqueous tear-deficient DE, showed lower band densities on MMP-9 immunoassay. Therefore, a highly inflamed ocular surface with decreased tear volume, such as that found in Sjögren syndrome, could show negative results because of the markedly decreased tear secretion, despite the highly elevated tear MMP-9 concentration. However, clinically, the opposite is possible. Among the participants of the present study, a strong positive band was identified even in patients with mild or nearly no fluorescein staining of the cornea and conjunctiva, who are expected to have very mild inflammatory eye surface inflammation.

Nevertheless, there was no difference in the strip meniscometry results between the MMP-9-positive and -negative groups. In particular, the MMP-9 test is usually performed first, after noninvasive tests, such as TMH, and thus, the volume of tears stored on the ocular surface and the amount of tear volume after stimulation of the strip meniscometry may be fundamentally different. In addition, the strip meniscometry was performed after the MMP-9 immunoassay, and recovery of tears depleted by the sampling apparatus of the MMP-9 immunoassay may take time. Since the low TMH group represents aqueous tear-deficient DE, subjects belonging to this group would require more time for recovery than those in the other groups. In terms of concentration, since MMP-9 is secreted not by the lacrimal gland, but by the ocular surface epithelium, reflex tearing may dilute the solutes dissolved in tears. Therefore, tear parameters that were measured by anterior OCT would better reflect the actual loading volume than the strip meniscometry in the MMP-9 immunoassay.

The present study has some limitations. As we only included participants who showed dry eye symptoms and signs at initial examination, there was no control group for comparisons of InflammaDry results with dry eye groups. Thus, further analysis between dry and not-dry eye patients would be helpful for understanding the clinical features of the InflammaDry test. In addition, because classification according to the tear meniscus value is very artificial, there may be significant limitations based on whether the three groups are appropriate. However, variability in TMH values may be inevitable because these can be very different depending on race, measurement equipment, and subtype of the dry eye.

In conclusion, we found the volume dependency of the MMP-9 immunoassay, which could induce false-negative results clinically. In particular, moderate to severe forms of aqueous tear-deficient DE conditions, such as Sjögren syndrome, could be underdiagnosed by the commercially available tear MMP-9 immunoassay. In addition, our results suggest that appropriate tear collection by the sampling fleece of the MMP-9 immunoassay is crucial to the appropriate detection of ocular surface inflammation.

## Supporting information

S1 File The raw excel file of dry eye parameters. (XLSX)
